# Identification and Characterization of the Gene *CYP340W1* from *Plutella xylostella* and Its Possible Involvement in Resistance to Abamectin

**DOI:** 10.3390/ijms17030274

**Published:** 2016-03-18

**Authors:** Xue Gao, Jiaqiang Yang, Baoyun Xu, Wen Xie, Shaoli Wang, Youjun Zhang, Fengshan Yang, Qingjun Wu

**Affiliations:** 1 Department of Plant Protection, Institute of Vegetables and Flowers, Chinese Academy of Agricultural Sciences, Beijing 100081, China; gaoxuexeu@163.com (X.G.); yjqhades@gmail.com (J.Y.); xubaoyun@caas.cn (B.X.); xiewen@caas.cn (W.X.); wangshaoli@caas.cn (S.W.); zhangyoujun@caas.cn (Y.Z.); 2 College of Life Science, University of Hei Longjiang, Harbin 150000, China; yangfshan@126.com

**Keywords:** *Plutella xylostella*, P450 *CYP340W1*, overexpression, abamectin resistance, RNA interference

## Abstract

Abamectin has been used to control the diamondback moth, *Plutella xylostella* (*P. xylostella*), which is a major agricultural pest that can rapidly develop resistance against insecticides including abamectin. Although cytochrome P450 has been confirmed to play an important role in resistance in *P. xylostella*, the specific P450 genes associated with the resistance are unclear. The full-length cDNA of the cytochrome P450 gene *CYP340W1* was cloned and characterized in the present study. The cDNA assembly yielded a sequence of 1929 bp, containing the open reading frame (ORF) 1491 bp and encodes a 496-amino acid peptide. *CYP340W1* was expressed in all *P. xylostella* developmental stages but its expression level was highest in larvae and especially in the heads of larvae. The expression of *CYP340W1* was significantly higher in an abamectin-resistant strain (ABM-R) than in its susceptible counterpart (ABM-S). In addition, expression of *CYP340W1* was increased when the ABM-R strain was exposed to abamectin. When injected into third-stage ABM-R larvae, *CYP340W1* dsRNA significantly reduced *CYP340W1* expression at 6 h and reduced expression by 83% at 12 h. As a consequence of RNAi, the mortality of the injected abamectin-resistant larvae increased after a 48-h exposure to abamectin. The results indicate that the overexpression of *CYP340W1* plays an important role in abamectin resistance in *P. xylostella.*

## 1. Introduction

The diamondback moth (DBM) *Plutella xylostella* (L.) (*P. xylostella*) is a serious cosmopolitan pest that can cause great damage, mainly to cruciferous crops. The total annual cost of damage caused by *P. xylostella* has been estimated to be US$4–5 billion [1]. *P. xylostella* is notorious for its rapid development of resistance to insecticides, including abamectin. Abamectin, which was developed from secondary metabolites of the soil bacterium *Streptomyces avermitilis*, is a mixture of avermectin B1a and avermectin B1b of the macrocyclic lactone group [2]. Avermectins have a broad spectrum of activity against crop pests, including mites and also insects in the orders Coleoptera, Homoptera, Diptera, Orthoptera, Isoptera, Hymenoptera, and Lepidoptera [3]. *P. xylostella* was highly susceptible to abamectin during the initial years of application, but abamectin resistance in *P. xylostella* was first found in 1996 in the Cameron Highlands in Malaysia [4]. Abamectin resistance in *P. xylostella* was subsequently reported in Brazil [5] and Pakistan [6]. A high level of resistance to abamectin in *P. xylostella* was found in southern China; in Yunnan Province, for instance, the resistance level was about 5000-fold [7]. Despite this, abamectin and its derivatives are still used to control *P. xylostella* and other pests in northern China and in other countries [8,9]. Managing abamectin resistance and prolonging the life of avermectins depends on understanding the resistance mechanism (s).

Researchers have studied the mechanisms underlying *P. xylostella* resistance to abamectin, and they have proposed many hypotheses. Previous studies indicated that abamectin resistance in *P. xylostella* is autosomal, incompletely dominant, and polygenic in both field and laboratory-selected strains [7,10]. Reduced cuticle penetration of insecticides [11], increased detoxification enzymes [12,13], target-site mutations [14], and ATP-binding cassette (ABC) transporters [15] have been reported to contribute to abamectin resistance in *P. xylostella*. The increased activity of cytochromome P450 monooxygenase was confirmed to play an important role in abamectin resistance in *P. xylostella* [7]. The role of P450s in insecticide resistance was first reported in the early 1960s when researchers found that the resistance of houseflies to carbaryl could be eliminated by the P450 inhibitor sesame [16]. Since then, P450s have been a focus of resistance research, and the evidence indicating that P450s mediate resistance has increased rapidly [17,18]. The up-regulation of P450 genes belonging to the families CYP6, CYP9, and CYP12 has been reported in resistant insects [19]. Overexpression of *CYP4G61* is associated with pyriproxyfen resistance in the greenhouse whitefly *Trialeurodes vaporariorum* [20]. Knockdown of the *CYP6BG1* gene in *P. xylostella* by RNA interference (RNAi) significantly reduced the resistance to permethrin [21]. High levels of abamectin resistance in *Tetranychus urticae* are associated with the metabolism of abamectin by *CYP392A16* [22]. The overexpression of the cytochrome P450 gene *Cyp12a4* is associated with lufenuron resistance in a natural population of *Drosophila melanogaster* [23]. *CYP9A12* and *CYP9A14* are associated with pyrethroid resistance in *Helicoverpa armigera* [24]. Finally, a recent report indicated that *CYP340* genes probably contribute to the detoxification of insecticides or plant toxins in *P. xylostella* [25].

Although P450s have been confirmed to be important for abamectin resistance in *P. xylostella*, the specific P450 genes associated with this resistance are unclear. We previously sequenced the transcriptomes of abamectin-susceptible and -resistant strains of *P. xylostella* using Illumina HiSeqTM 2000. We found that only one gene among the large P450 family was significantly up-regulated in the resistant strain (unpublished data). In the current study, we cloned and characterized this gene and investigated its function. Our objective was to clarify the characteristic of *CYP340W1* gene in *P. xylostella* population and investigate role of *CYP340W1* in the abamectin resistance of *P. xylostella*.

## 2. Results

### 2.1. Cloning and Characterization of the CYP340W1 Gene

**Figure 1:**
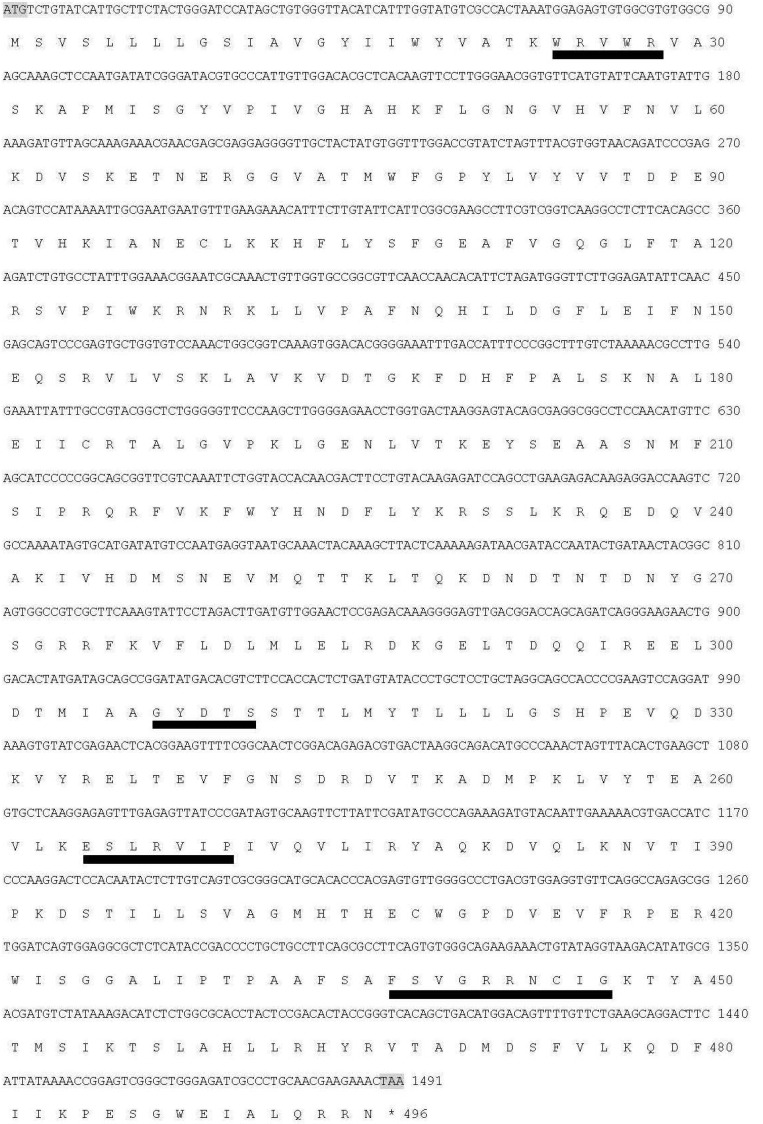
Nucleotide sequence and deduced amino acid sequence of CYP340W1 in *P. xylostella*. The following P450 signature motifs are underlined in black: helix C motif (position 24), the oxygen-binding motif (helix I) (position 307), the helix K motif (position 363), and the heme-binding motif (position 437). The initiation and termination codons are shaded gray color. * indicates stop codon.

The full-length sequence of the P450 gene was obtained by RT-PCR and RACE methods based on the partial sequence in the unpublished transcriptome database. The cDNA sequences were submitted to the P450 nomenclature committee, who assigned the gene to the CYP340 family and named it *CYP340W1*. The full-length cDNA of *CYP340W1* includes a 1491-bp open reading frame (ORF), a 299-bp 5’-untranslated region (UTR) containing a TATA box, and a 139-bp 3’UTR containing a 26-bp poly-A tail. The predicted isoelectric point of the protein is 9.54, and the theoretical molecular weight is 56.29 kDa. The *CYP340W1* cDNA encodes 496 amino acids. The amino acid sequence contains conserved domains characteristic of P450s such as the helix C motif (WxxxR, position 24), the oxygen-binding motif (helix I) (GxDTS, position 307), the helix K motif (ExxRxxP, position 363), and the heme-binding motif (FxxGxxxCxG, position 437) (Figure 1).

### 2.2. Expression Profile of CYP340W1 in Different Developmental Stages and Tissues of P. xylostella

**Figure 2:**
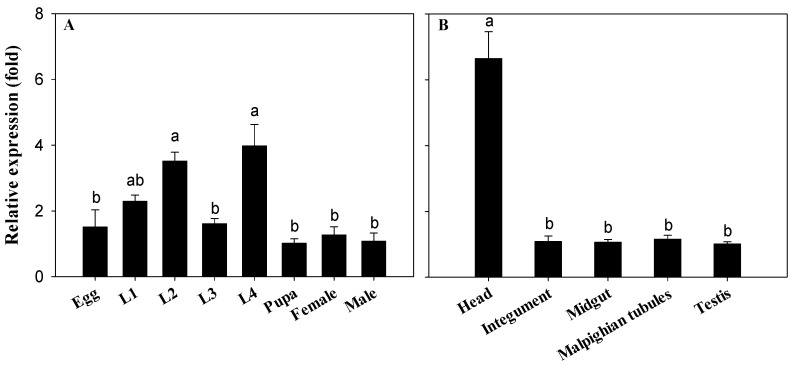
Expression profiles of *CYP340W1* in different developmental stages (**A**) and tissues of the fourth instars (**B**) of ABM-S. L1 to L4: the first- to fourth- instar larva. Expression in pupa (**A**) and midgut (**B**) was set to 1. Values are means ± SEs for three independent replicates. In each panel, means with different letters are significantly different (*p* < 0.05; Tukey’s test).

Susceptible strain of *P. xylostella* was used to measure the *CYP340W1* expression at different developmental stages and the expression profiling at tissue level was for the fourth instars. *CYP340W1* was expressed during the entire life cycle of *P. xylostella* (Figure 2A). *CYP340W1* levels were significantly higher in the second- and fourth-instar larvae than in other developmental stages (*p* < 0.05; Tukey’s test). The relative expression level of *CYP340W1* was about six-fold higher in the head than in the other tissues (Figure 2B). The expression level was similar in tissues of the integument, midgut, malpighian tubules, and testis.

### 2.3. Transcriptional Response of CYP340W1 to Abamectin Exposure

**Figure 3:**
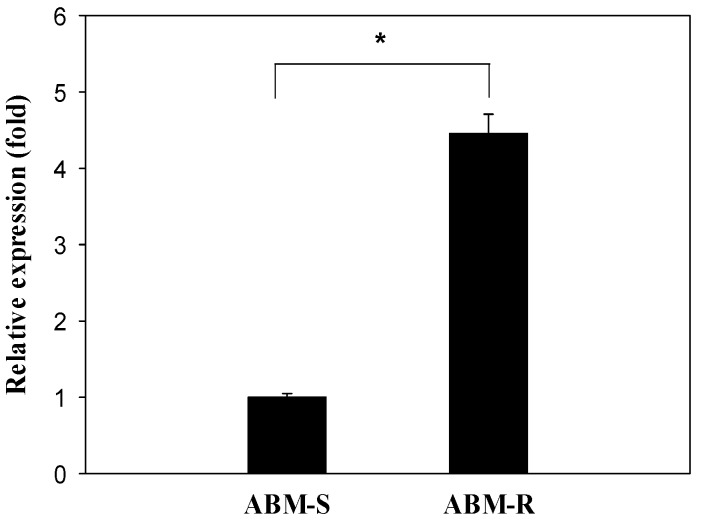
The relative expression of *CYP340W1* in the fourth instars of ABM-S and ABM-R in the absence of abamectin treatment. Expression in the ABM-S strain was set to 1. Expression of the elongation factor 1 (*EF1*) and ribosomal protein L32 (*RPL32*) genes were used as the internal standard. Values are means and standard errors. The asterisk indicates a significant difference between the ABM-S and ABM-R strain (*p* < 0.05; Tukey’s test; *n* = 3).

**Figure 4:**
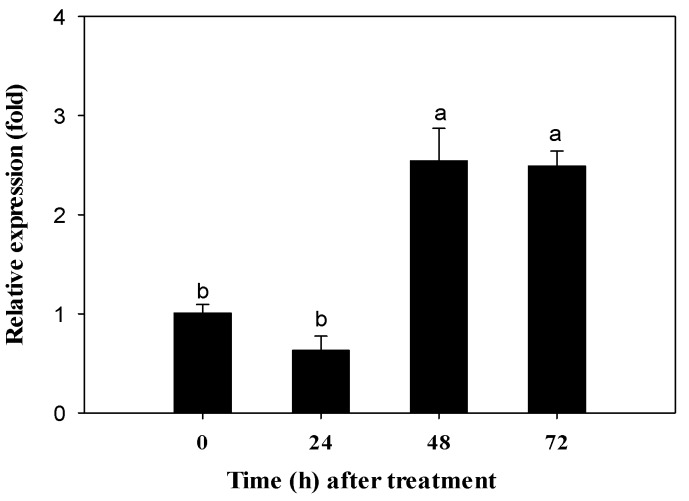
Relative gene expressions of *CYP340W1* in ABM-R after the third instars were treated with LC_10_ concentration of abamectin for different time. Expression at 0 h was set to 1. Data are presented as means ± SE. The lowercase letters above the error bars mean significant difference in expression levels according to Tukey’s test *p* < 0.05 (*n* = 3).

The resistance ratio of the ABM-R strain was about 115-fold relative to the ABM-S strain (Table 1). In the absence of abamectin treatment, the *CYP340W1* level was significantly higher in the ABM-R strain than in the ABM-S strain (Figure 3). To investigate the effect of abamectin on *CYP340W1* in *P. xylostella*, the third-instar larvae of ABM-R were treated with abamectin at 0.4 mg/L, which is the LC_10_ value for the ABM-R strain (Table 1). The *CYP340W1* expression level had increased significantly after 48 h with abamectin treatment (Figure 4). The most of mortality was realized within 48 h.

### 2.4. Expression of CYP340W1 after RNAi

**Figure 5:**
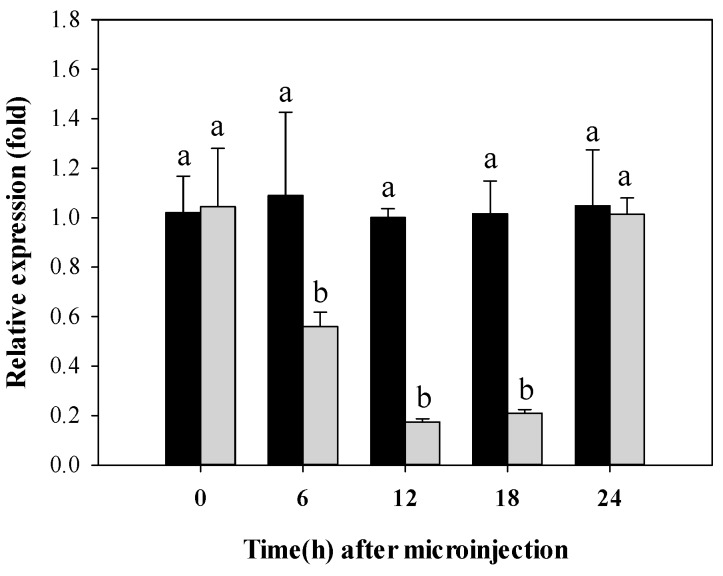
Relative expression levels of *CYP340W1* (grey bars) at different times after *CYP340W1* dsRNA was injected into third-stage larvae of ABM-R. Expression is relative to that of *CYP340W1* injected dsEGFP (black bars), and *CYP340W1* expression injected dsEGFP was set to 1. Values are means and standard errors. The lowercase letters above the error bars mean significant difference in expression levels according to Tukey’s test *p* < 0.05 (*n* = 3).

To investigate the efficiency of RNAi knockdown of *CYP340W1* in third-instar larvae of *P. xylostella*, the levels of *CYP340W1* mRNA were measured with qRT-PCR at 0 to 24 h after the larvae had been injected with *CYP340W1* dsRNA. Relative to *CYP340W1* expression levels injected with dsEGFP, the *CYP340W1* expression levels injected with dsCYP340W1 were significantly reduced at 6, 12, and 18 h but not at 24 h after dsRNA injection (Figure 5).

### 2.5. Effect of CYP340W1 Silencing on Larval Mortality

**Figure 6:**
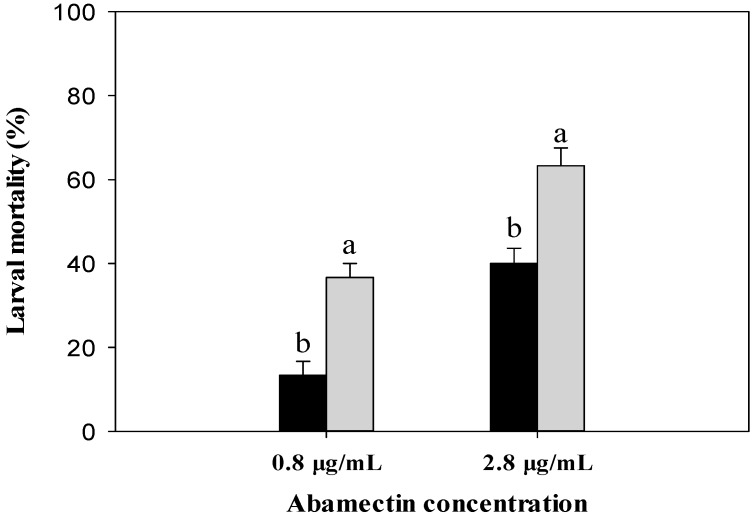
Mortality of strain ABM-R after the third instars were injected with dsRNA targeting CYP340W1 (grey bars) or with dsRNA targeting EGFP (black bars) and then treated with one of two concentrations of abamectin. The larvae were treated with abamectin 6 h after they had been injected with the dsRNA, and mortality was assessed 48 h later. Values are means and standard errors. Means with different letters are significantly different (*p* < 0.05; Tukey’s test; *n* = 3).

Third-stage larvae of ABM-R were placed in abamectin solutions 6 h after they had been injected with dsCYP340W1 or dsEGFP, and their mortality was assessed 48 h later. With both concentrations of abamectin, mortality was significantly higher after injection with dsCYP340W1 than after injection with dsEGFP (Figure 6). About 36% mortality was observed in larvae injected with dsCYP340W1 treated with 0.8 μg/mL (LC_20_) of abamectin, while only 13% mortality was observed in control larvae injected with dsEGFP. When the abamectin concentration was 2.8 μg/mL (LC_50_), about 63% mortality was detected in larvae injected with dsCYP340W1, while 40% mortality was observed in control.

## 3. Discussion

Abamectin has been used to control *P. xylostella* for more than 20 years, and many *P. xylostella* populations worldwide have developed resistance to the insecticide [4,26,27]. Although cytochrome P450-mediated metabolic resistance is recognized as important in the resistance of *P. xylostella* [21,28], no cytochrome P450 gene from *P. xylostella* had been reported to be responsible for abamectin resistance before the current study. From our transcriptome database, we found that the expression of only one P450 gene was higher in abamectin-resistant than in abamectin-susceptible *P. xylostella* (unpublished dada). In the present study, the full-length cDNA of this gene was obtained by RT-PCR and RACE methods and was named *CYP340W1* by the Nomenclature Committee.

P450 gene overexpression usually has two kinds of mechanism, on the one hand, genetic mutation gene promoter or enhancer [20], on the other hand, is caused by gene amplification quantity increases [29]. We used qRT-PCR to investigate the expression profile of *CYP340W1*. The results showed that *CYP340W1* is expressed in all developmental stages of *P. xylostella* and that expression is highest in larvae. The expression of another P450 gene, *CYP6A1*, in the housefly is high in both larvae and adults but low in pupae and eggs [30]. In the Asian corn borer, *CYP6AE25* is expressed in all life stages [31]. The current results suggest that cytochrome P450 *CYP340W1* is more important in larvae than in eggs, pupae, or adults. It is well known that insect P450s are expressed in various tissues when the insects are exposed to diverse physiological and environmental stimuli [32]. Unlike other P450 genes that are highly expressed in the midgut [33,34], which is regarded as the primary detoxification organ, *CYP340W1* is much more abundant in the head than in other tissues. The P450 gene *CYP4G15* is also primarily expressed in the brain of third instars of *Drosophila* [35]. Abamectin is a neurotoxin insecticide that interferes with neural and neuromuscular system mostly by enhancing the glutamate-gate chloride channel [36]. The high expression of *CYP340W1* in the heads of *P. xylostella* larvae might provide the last line of defense to protect the target site [37]. Zhu *et al.* found that the P450 gene *CYP6BQ9* was predominantly expressed in the brain and was responsible for deltamethrin resistance in *Tribolium castaneum*. The authors suggested that the brain-specific expression of this gene could enhance the ability of brain cells to metabolize deltamethrin and to thus reduce the levels of the insecticide at the target site [38].

We found that the expression of *CYP340W1* was significantly higher in an abamectin-resistant strain than in an abamectin-susceptible strain of *P. xylostella*. This is consistent with many reports that insecticide resistance is related to the overexpression of P450 genes [32,39,40]. Induced expression is an important way to study the insecticide resistance that is caused by detoxification enzymes. In *Drosophila*, for example, mutation resulting in the higher expression of an inducible cytochrome P450 gene was thought to be a likely way in which pesticide resistance developed [41]. The P450 gene *CYP6BG1* in permethrin-resistant *P. xylostella* can be induced by a low dose of permethrin [42]. In the present study, *CYP340W1* in the resistant strain was significantly induced by a low dose of abamectin. This induction suggested that *CYP340W1* might be involved in abamectin resistance in *P. xylostella*. Because RNAi has been widely used to investigate gene functions in insecticide-resistant insects, we injected dsRNA of *CYP340W1* into the third-instar larvae of the resistant strain and found that the expression level of *CYP340W1* was significantly decreased at 6 h and was lowest at 12 and 18 h post injection. Based on previous reports, RNAi reduction in the expression level of P450 genes usually requires 24 h [21,28], *i.e.*, the reduction documented in the current study occurred sooner than previously reported for other P450 genes in *P. xylostella*. We then found that larval mortality caused by abamectin in the abamectin-resistant strain of *P. xylostella* was significantly increased after *CYP340W1* was silenced, strongly indicating that this gene is involved in abamectin resistance in *P. xylostella*.

## 4. Materials and Methods

### 4.1. Insects

A susceptible strain of *P. xylostella* (ABM-S) was originally collected in 1990 from a cabbage (*Brassica* sp.) field in Guangzhou, Guangdong Province, China, and has since been reared in the laboratory without further exposure to insecticide. The abamectin-selected strain (ABM-R) consisted of the offspring of ABM-S that had been selected with abamectin. The *P. xylostella* larvae were reared on cabbage plants under conditions of 25 ± 1 °C, 60%–70% relative humidity and a light: dark cycle of 16:8 h. Adults were provided with a 10% honey solution.

### 4.2. Chemicals and Bioassay

Abamectin (containing 93% avermectin B1a and 7% avermectin B1b) was obtained from the Department of Applied Chemistry, China Agricultural University. The leaf-dipping method (in which larvae are placed on segments of cabbage leaves that have been dipped into abamectin solutions) was used for the bioassay as described by Liu *et al.* [43] to establish LC_50_, LC_20_, and LC_10_ values. Five series of concentrations was set and each concentration was performed in three replicates with ten 3rd instar larvae for each replicate. For ABM-R strain the concentration ranged from 0.625 to 10 μg/mL, and for ABM-S strain the concentration ranged from 0.00625 to 0.1 μg/mL. Mortality was assessed at 48 h post-treatment. The Poloplus program (LeOra Software 2002) was used for probit analysis of the dose data.

### 4.3. Molecular Cloning of *CYP340W1*

Total RNA of the *P. xylostella* was exacted from the fourth instar larvae by using TRIzol reagent (Invitrogen, Carlsbad, CA, USA) following the manufacturer’s protocol. First-strand cDNA was synthesized with the PrimeScript II 1st strand cDNA synthesis kit with oligo dT primer (Takara Biotechnology, Dalian, China). Rapid amplification of cDNA ends (RACE) with SMARTer RACE cDNA Amplification (Clontech, Palo Alto, CA, USA) kits was used to obtain the full-length cDNA of *CYP340W1* following the manufacturer’s instructions. Gene-specific primers (GSPs) were designed with Primer Premier 5.0 (Premier Biosoft International, Palo Alto, CA, USA), and the RACE primers were listed in Table 2. The 3’ RACE was performed directly using 3’ GSP1 and the universal adaptor primer (UPM). The PCR reactions (25 μL total volume) contained 15.5 μL of double-distilled H_2_O (ddH_2_O), 2.5 μL of 10× LA Taq Buffer, 2 μL of dNTP Mix, 1 μL of specific primer, 1.5 μL of first-strand cDNA template, 2 μL of UPM, and 0.5 μL of Advantage2 Taq HS (TaKaRa). The touchdown PCR reaction conditions were: five cycles of 94 °C for 30 s and 72 °C for 3 min; followed by five cycles of 94 °C for 30 s, 68 °C for 30 s, and 72 °C for 3 min; and finally 25 cycles of 94 °C for 30 s, 66 °C for 30 s, and 72 °C for 3 min. In 5’RACE, the PCR reactions contained 15.7 μL of ddH_2_O, 2.5 μL of 10× LA Taq Buffer, 2 μL of dNTP Mix, 1 μL of specific primer, 2 μL of UPM, 1.5 μL of first-strand cDNA template, and 0.3 μL of LA Taq HS polymerase. The PCR reaction conditions were: five cycles of 94 °C for 30 s and 72 °C for 3 min; followed by five cycles of 94 °C for 30 s, 66 °C for 30 s, and 72 °C for 3 min; and finally 25 cycles of 94 °C for 30 s, 62 °C for 30 s, and 72 °C for 3 min. To verify the full-length of P450 cDNA, a pair of primers (CYP-Full-F and CYP-Full-R, Table 2) was used to amplify the open reading frame (ORF) of *CYP340W1* cDNA. The PCR reactions (25 μL total volume) contained, 2.5 μL of 10× LA Taq Buffer, 2 μL of dNTP Mix, 1 μL of first-strand cDNA template, 1 μL of specific primer, 0.25 μL LA Taq HS polymerase (TaKaRa), and 18.25 μL of double-distilled H_2_O (ddH_2_O). All PCR reactions were carried out with a S1000 PCR Thermal Cycler (Bio-Rad, Shanghai, China). PCR was performed using the following program: an initial denaturation at 94 °C for 3 min, followed by 35 cycles of 94 °C for 30 s, 56 °C for 30 s, and 72 °C for 2 min and a final extension at 72 °C for 10 min.

The open reading frame (ORF) was predicted using the ORF Finder tool at the NCBI website (http://www.ncbi.nlm.nih.gov/guide/all/#tools_). The gene sequence was assembled with DNAMAN 7.0 (Lynnon BioSoft, San Ramon, CA, USA). The cDNA sequence was translated into an amino acid sequence with the translate tool at the ExPASy proteomic website (http://www.expasy.org/tools).

### 4.4. Quantitative RT-PCR

Using quantitative real-time reverse transcription polymerase chain reaction (qRT-PCR), we measured the relative expression levels of *CYP340W1* in larvae of ABM-S and ABM-R strains. Relative expression levels of *CYP340W1* were also measured in different developmental stages and in different tissues of fourth-instar ABM-S larvae. Regarding expression in different developmental stages including eggs, larvae (first to fourth instars), pupae, and female and male adults with one day old were used Regarding expression in different tissues, the head, integument, midgut, malpighian tubules, and testis of fourth-instar larvae were dissected and placed in PBS (137 mM/L NaCl, 2.7 mM/L KCl, 10 mM/L Na_2_HPO_4_, and 2 mM/L KH_2_PO_4_). Abamectin induction of the *CYP340W1* gene was measured by the leaf-dipping method, *i.e.*, third-instar larvae of the ABM-R strain were placed on segments of cabbage leaves that had been dipped in a solution containing 0.4 mg of abamectin/L (the LC_10_); *CYP340W1* induction was measured by qRT-PCR after the larvae had been on the leaves for 1, 2, and 3 days. Dissociation curve of four replicates using five 2-fold serial dilutions (1:1, 1:2, 1:4, 1:8, and 1:16) were analyzed to obtain the amplification efficiencies and linear correlation between the quantity of cDNA templates and the quantity of PCR product, which was generated by the gene-specific primers. Screening all the results, only those with single-peak melting curve and 95%–100% primer amplification efficiencies were adopted. Four technical replicates and three biological replicates were used for each treatment.

After total RNA for RT-qPCR was extracted using TRIzol reagent (Invitrogen), 1 μg was used to prepare the first-strand cDNA using the PrimeScript RT kit (containing gDNA Eraser, Perfect Real Time) (TaKaRa) following the manufacturer’s instructions. qRT-PCR was performed with an ABI PRISM 7500 Real-time PCR System (Applied Biosystems, Foster, CA, USA) using the SuperReal PreMix Plus (SYBR Green) kit (Tiangen, Beijing, China). The primers CYP340-QF and CYP340-QR (Table 2) were designed with Primer Premier 5 software to specifically amplify a 160-bp fragment of the *CYP340W1* gene. The primer elongation factor 1 gene (*EF1*) accession number EF417849 and ribosomal protein L32 gene (*RPL32*) accession number AB180441 (Table 2) were used as reference genes [44]. Normalizing the CYP340W1 expression with two housekeeping genes was performed according to Vandesompele *et al.* (2002) [45]. PCR reactions (20 μL) contained 2 μL of cDNA, 0.5 μL of forward primer, 0.5 μL of reverse primer, 10 μL of 2× SuperReal PreMix Plus, 0.4 μL of 50× ROX Reference Dye, and 6.6 μL of RNase-free ddH_2_O. The thermal cycling conditions included polymerase activation at 95 °C for 15 min followed by 40 cycles of denaturation at 95 °C for 10 s, annealing at 60 °C for 32 s, and elongation at 72 °C for 32 s. The melt curve analysis included a final step at 95 °C for 15 s and 60 °C for 60 s. The RT-qPCR analysis included three biological replicates and four technical replicates for each treatment. The relative abundance of gene transcripts was calculated according to the 2^−∆∆*C*t^ method [46].

### 4.5. dsRNA Synthesis

A double-stranded RNA (465 bp) corresponding to a portion of the *CYP340w1* gene was synthesized. The amplicon was used as template for *in vitro* transcription reactions to generate dsRNA using the T7 Ribomax Express RNAi System (Promega, Madison, WI, USA). The *P. xylostella* genome was blasted and designed to the specific region of dsRNA to avoid potential off-target effects. The SnapDragon tool (http://www.flyrnai.org/cgi-bin/RNAi_find_primers.pl) was used to design specific primers as shown in Table 2. The PCR products of 465 bp were examined on agarose gel before *in vitro* transcription to verify that the products consisted of a single band of the expected sizes. *EGFP* was used as reference gene, and the primers for dsEGFP were used according to Guo *et al*. (2015) [47]. Injection was performed with the volume ratio 1:1 of Metafectene PRO transfection reagent (Biontex, Planegg, Germany) Mixed at 25 °C after incubation for 20 min to increase dsRNA stability and facilitate dsRNA delivery. All dsRNA preparations were quantified and then stored at −20 °C.

### 4.6. RNAi Assays

Expression of the *CYP340W1* gene was silenced by injecting *CYP340W1* dsRNA into the early third-instar of *P. xylostella* larvae of the ABM-R strain. Sterilized fine glass capillary microinjection needles was pulled by P-97 micropipette puller (Sutter Instrument, Novato, CA, USA), which was used to deliver 70 nL of injection dsCYP340W1 (300 ng) or dsEGP (containing Metafectene PRO solution) into the hemocoel of each larva. The injection was carried out with an SXZ10 microscope and nanoliter 2000 microinjection system (World Precision Instruments Inc. Sarasota, FL, USA) The treated larvae were starved for 6 h and anesthetized for 30 min on ice before injection. The experiment was performed for three times and over 20 larvae were injected for each time to investigate effectiveness of RNAi. At 0 to 24 h post-injection, total RNA was isolated from the specimens as described earlier, and tested expression of *CYP340W1* by qRT-PCR.

The effect of RNAi resulting from injection of dsCYP340W1 or dsEGFP was assessed by measuring mortality of injected larvae after they were exposed to abamectin via the leaf-dip bioassay; the leaf-dip bioassay was initiated 6 h after dsRNA injection and used abamectin concentrations approximating the LC_20_ value (0.8 μg/mL) and LC_50_ value (2.8 μg/mL). The leaf-dip assays were terminated after 48 h. Bioasssays were performed with thirty larvae per RNAi treatment and abamectin concentration, and each bioassay replicated three times.

### 4.7. Statistical Analysis

Results are presented as means and standard errors. The means were separated by Tukey’s test at *p* < 0.05 using SPSS 19.0 for Windows (SPSS Inc., Chicago, IL, USA).

## 5. Conclusions

In conclusion, we identified and characterized a cytochrome P450 gene, *CYP340W1*, in *P. xylostella*. The expression of *CYP340W1* was significantly up-regulated in an abamectin-resistant strain of *P. xylostella*. *CYP340W1* was induced by a low dose of abamectin. Microinjection of dsRNA of *CYP340W1* significantly reduced *CYP340W1* expression and significantly increased the susceptibility of resistant larvae to abamectin. Overall, the results suggest that overexpression of *CYP340W1* plays an important role in abamectin resistance in *P. xylostella*.

## Figures and Tables

**Table 1:** Susceptibility of *P. xylostella* strains to abamectin treatment.

Strain	LC50 (mg/L) (95% FL)	Slope ± SE	Χ^2^ (df)	RR ^a^	LC_10_ (95% FL) (mg/L)	LC_20_ (95% FL) (mg/L)
ABM-S	0.024 (0.020–0.028)	1.862 ± 0.335	2.856 (3)	1	0.004 (0.001–0.009)	0.008 (0.013–0.015)
ABM-R	2.822 (2.013–4.920)	1.485 ± 0.257	5.529 (3)	114.96	0.478 (0.136–0.802)	0.879 (0.399–1.285)

^a^ LC_50_: median lethal concentration; FL: 95% fiducial limits; SE: standard error; RR: resistance ratio; LC_50_ of ABM-R/LC_50_ of ABM-S; Χ^2^ is Chi square and df is degree of freedom.

**Table 2:** Primers used for cloning and expression analysis of *CYP340W1*.

Experiment	Primer Name	Primer Sequence (5’-3’)
3’RACE	CYP-RACE3’	CGGCTACGACACGTCTTCCACCACT
5’RACE	CYP-RACE5’	GAACCGCTGCCGGGGGATGCTGAAC
Full-length confirmation	CYP-Full-F	CATCAATGTCTGTATCATTGCTTCT
	CYP-Full-R	TCTAGAAACATATTAATTAACAGGC
qRT-PCR	CYP340-QF	GTTTTCGGCAACTCGGACAG
	CYP340-QR	TGGGGATGGTCACGTTTTTC
Reference gene	EF1-F	GCCTCCCTACAGCGAATC
	EF1-R	CCTTGAACCAGGGCATCT
	RPL32-F	CCAATTTACCGCCCTACC
	RPL32-R	TACCCTGTTGTCAATACCTCT
